# *Mytilus galloprovincialis* as a Biomarker for Personal Care Product (PCP) Ingredients and UV Filters (UVFs) in Tunisian Coastal Waters: Correlation with the Chemical Composition of Polluted Seawater

**DOI:** 10.3390/toxics13100847

**Published:** 2025-10-06

**Authors:** Emna Nasri, Elhem Bouchiba, Bouthaina Brahmi, Siwar Bouyahi, Eduardo Alberto López-Maldonado, Mohamed Ali Borgi

**Affiliations:** 1 Laboratory of Biotechnology and Biomonitoring of the Environment and Oasis Ecosystems (LBBEEO), Faculty of Sciences of Gafsa, University of Gafsa, University Campus of Zarroug, Gafsa 2112, Tunisia; 2 Faculty of Sciences of Tunis, University of Tunis El Manar, Tunis 1068, Tunisia; 3 Faculty of Chemical Sciences and Engineering, Autonomous University of Baja California, Tijuana 22424, Mexico

**Keywords:** personal care product ingredients, UV filters, *Mytilus galloprovincialis*, biomarker, LC-MS/MS, toxicity, risk assessment

## Abstract

Today, the abundance of personal care product (PCP) ingredients and UV filters (UVFs) in coastal marine environments is a growing concern worldwide. In addition, mussels are the most commonly used sentinel organisms in bio-monitoring programs. In the current study, we collected mussels (*Mytilus galloprovincialis*) (over 6 months) from three seawater sites in Tunisia (Monastir, Sousse, and Mahdia). Analysis of the samples by liquid chromatography coupled to tandem mass spectrometry (LC-MS/MS) revealed the presence of 13 compounds among the 18 PCP ingredients and UVFs investigated. Avobenzone (AVO) and tert-butyl hydroxyphenyl benzotriazole (TBHPBT) were the most frequently observed, ranging from 121.076 ± 1.6 to 193.481 ± 5.5 ng g^−1^ and 20.987 ± 0.7 to 26.704 ± 1.7 ng g^−1^, respectively, with maximum values in the city of Sousse. 4-Hydroxybenzophenone (4HB) and benzophenone-1 (BP1) were also found in all mussel samples with levels in the range of 26.745 ± 0.4 ng g^−1^ and 12.53 ± 0.5 ng g^−1^, respectively. We observed a positive correlation with the chemical characterization of the contaminated seawater. The environmental hazards of PCP ingredients were estimated with the aim of performing a preliminary risk assessment at the environmental level. For this purpose, the estimated daily intake (EDI) of a substance was calculated. The results obtained revealed a high value of up to 68.36 ng kg body-weight^−1^ day^−1^. The high concentration observed in the samples reported for the target PCP ingredients could be partly attributed to their inefficient removal before being released into the sea. We now know that PCP ingredients and UVFs cause irreparable damage to coastal ecosystems and pose the greatest risk to the aquatic organisms tested.

## 1. Introduction

In recent years, polar compounds, such as personal care product (PCP) ingredients, have been widely used for beauty and hygiene purposes. It has been demonstrated that using these compounds can cause significant chemical releases into coastal seas, either directly through bathing or indirectly via air deposition and wastewater treatment plants (WWTPs) [1]. However, their behavior and fate in the environment are not fully understood, and there is little data on their toxic effects. According to the European Parliament (EP) (2009), PCP ingredients are a wide range of products often used by people for hygienic, medical, or cosmetic purposes. In recent decades, the innovation of PCPs has been considered hazardous to aquatic life [2], and PCP ingredients are commonly discharged from effluent treatment units [3]. PCP ingredients are very complex products of emerging contaminants, and their adverse effects have attracted the attention of researchers to evaluate their occurrence [4]. Hence, new ingredients have been introduced in recent years, mainly due to the socio-economic configuration of advanced societies. PCP ingredients are routinely released in large quantities and are poorly degradable in the environment [1]. In addition, they have been characterized by their biological activities and tendency to bioaccumulate in sediments and sludge [5], as well as in biota [6,7] and humans [8]. We anticipate that these compounds, usually with log Kow 4–8 (decimal logarithm of the octanol/water partition coefficient), will be able to reach and accumulate in aquatic species at varying trophic levels. While UV filters (UVFs) are intended to be applied externally to the skin or hair, some of them have the potential to enter the body, undergo further metabolism, and then become bioaccumulated or excreted [1]. Residues of pharmaceuticals, pesticides, nanomaterials, ingredients of PCPs, and many other man-made substances that reach the sea directly or indirectly are not adequately investigated and are mostly unregulated. Overall, the controlled and uncontrolled release of these chemicals poses a real threat to human health and the environment. Pollutants released into the environment whose effects are not well understood and not regulated are called emerging pollutants. These pollutants are not necessarily persistent, but they can cause problems because they are continuously released into the ecosystem, and for this reason, they are considered pseudo-persistent [9]. Many works have highlighted the significant impacts of PCP ingredients in altering the endocrine system and blocking or disrupting hormonal functions, even at low concentrations [10]. PCP ingredients enter marine waters mainly via WWTPs, especially in countries with the highest population density. Another important pathway is direct introduction, for example, by beachgoers, especially in summer due to nautical tourism [11]. UVFs, both organic and inorganic, and parabens (PBs) are of specific interest because of their toxic effects. It is worth noting that nanotechnology is now being used in the production of PCPs, e.g., in sunscreens [11]. Wastewater and many surface and groundwaters typically contain several UVFs and combined effects in contaminated ecosystems. In this context, chemicals are likely to co-occur in these areas [12]. They have been classified as pseudo-persistent compounds due to their poor biodegradability in the environment [13]. Their presence, ranging from µg L^−1^ to low ng L^−1^, has been documented in groundwater [14], wastewater treatment units [15], rivers [16], lakes [17], seawater [18], and hospital wastewater [19]. Recent literature on coastal ecosystems has documented the presence of PCP ingredients in seawater worldwide [20], but this has not been fully investigated. It is well documented that marine areas are under increasing pressure from higher population densities and marine tourism. However, it should be noted that the potential bioaccumulation and biomagnification of certain PCP ingredients may be responsible for greater ecotoxicity in the marine food web [21]. It is therefore urgent to pay attention to the impact of PCP ingredients on marine ecosystems and to identify vulnerable coastal areas. Following this idea, the main goal of the current work is to carry out the quantification of PCP ingredients and UVFs in seawater and mussels (*Mytilus galloprovincialis*) in three Tunisian coastal areas (Sousse, Monastir, and Mahdia).

## 2. Materials and Methods

### 2.1. Reagents and Chemicals

The chemical standards 2,4-dihydroxybenzophenone (BP1, ≥99% purity, Fluka, Buchs, Switzerland); 2,2′,4,4′- tetrahydroxybenzophenone (BP2, 97% purity, Sigma Aldrich, St. Louis, United states of America (USA)); 2-hydroxy-4- methoxybenzophenone (BP3, 98% purity, Sigma Aldrich, St. Louis, USA); 4-hydroxybenzophenone (4HB, 98% purity, Fluka, Buchs, Switzerland); 4,4′- dihydroxybenzophenone (4DHB, 99% purity, Sigma Aldrich, St. Louis, USA); 2,2′-dihydroxy-4- 14 Vilaró Ruiz, Gemma methoxy-benzophenone (DHMB, 98% purity, Sigma Aldrich, St. Louis, USA); avobenzone (AVO, 99% purity, Sigma Aldrich, St. Louis, USA); 2-ethylhexyl-trans-4-methoxycinnamate (EHMC, 98% purity, Sigma Aldrich, St. Louis, USA); 3-(4-methylbenzyliden) camphor (4MBC, ≥98% purity, Sigma Life Science, St. Louis, USA); 2-ethylhexyl 4- (dimethyl-amino) benzoate (OD-PABA, 98% purity, Merck, Darmstadt, Germany); Ethyl-4-aminobenzoate (Et-PABA, 98% purity, Sigma Aldrich, St. Louis, USA); Ethyl 4-aminobenzoate (MeBZT, ≥99% purity, TCI (Tokyo Chemical Industry), Tokyo, Japan); 2-(5-tert-butyl-2- hydroxyphenyl)benzotriazole (TBHPBT, >98% purity, TCI, Tokyo, Japan); 2-(2-benzotriazolyl)-p-cresol (UVP, >98% purity, TCI, Tokyo, Japan); 2-(2H-benzotriazole-2-yl)-4,6-bis(1-methyl-1-phenylenthyl) phenol (UV234, >98% purity, TCI, Tokyo, Japan); 2-(3,5-di-tert-butyl-2-hydroxyphenil)-5-chlorobenzotriazole (UV327, >98% purity, TCI, Tokyo, Japan); 2-(3,5-di-tert-amyl-2-hydroxyphenyl) benzotriazole (UV328, 98% purity, TCI, Tokyo, Japan); 5,6-dimethyl-1H-benzotriazole monohydrate (DMeBZT, 99% purity, TCI, Tokyo, Japan). The isotopically labeled compounds used as internal standards, 3-(4-methylbenzylidined4)camphor (4MBC-d4), 2-hydroxy-4-methoxybenzophenone-(phenyl-d5) (BP3-d5), and 2-(2Hbenzotriazol-2-yl)-4-methyl-6-(2-propenyl)phenol (AllylBZT), were obtained from Carbon, Deuterium, and Nitrogen (CDN) isotopes (from Quebec, Canada). Acetonitrile (ACN), methanol (MeOH) and formic acid, and HPLC-grade water were provided by John Townsend (J.T.) Baker Chemical Company, Easton, Pennsylvania, USA. The standard solutions were prepared by dissolving the following pure compounds and taking the appropriate aliquot to the desired concentrations. The internal standards-deuterated (ISD) were prepared from individual standard solutions made up to 500 ppb. The calibration standards were prepared from individual standard solutions taken to 100 ppm. These were diluted to concentrations of 1, 3, 5, 10, 30, 50, 100, 300, and 500 ppb for calibration. Working solutions were prepared by diluting standard solutions through weighing. All solutions were kept in glass vials in a refrigerator at 4 °C until use. The chemical standards analyzed in the present study, with their corresponding INCI (International Nomenclature Cosmetic Ingredients) names and function in cosmetic products, are illustrated in Table S1.

### 2.2. Sampling

**Figure 1:**
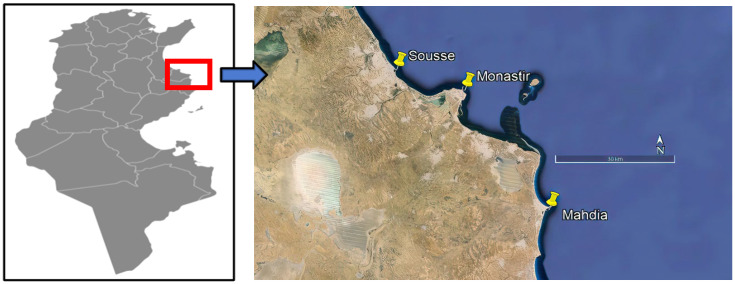
Geographical location of the sampling sites of mussels and seawater in coastal areas from the governorates of Sousse (latitude 35°49′30.36″ N; longitude 10°38′58.96″ E), Monastir (latitude 35°46′37.81″ N; longitude 10°50′21.06″ E), and Mahdia (latitude 35°29′48.53″ N; longitude 11°4′19.04″ E) in Tunisia.

Seawater and mussel (*Mytilus galloprovincialis*) samples were collected from the Sahel region of central eastern Tunisia, specifically from the governorates of Sousse, Monastir, and Mahdia (Figure 1). These sites were selected due to their economic activities in tourism and aquaculture. At each site, samples were collected from three locations (5 m spots), situated approximately 10 km from municipal wastewater treatment plants. Sampling was performed monthly from March to August 2019, with three replicates at each site.

- Seawater: Collected from the upper water column (≈30 cm) in 1 L glass bottles.
- Mussels: Collected using a benthic grab sampler. To reduce variability (size, age, environmental conditions, and health status), 10 mussels of a similar size were pooled per sample, resulting in 30 composite samples per month.

**Figure 2:**
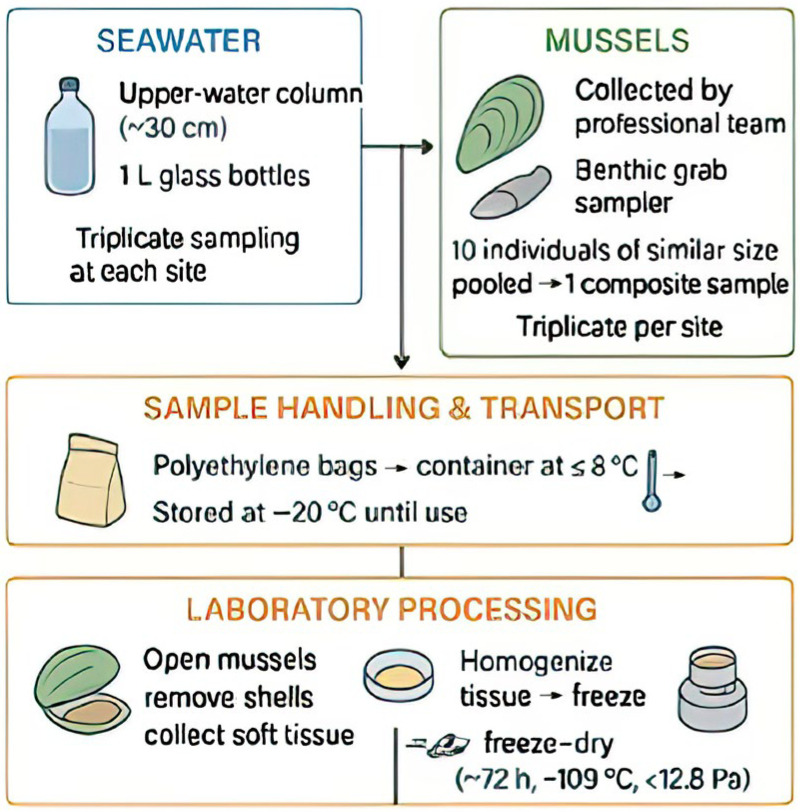
Steps illustrating the sampling procedures of seawater and mussels.

The samples were then transported in polyethylene bags at ≤8 °C to the laboratory and stored at −20 °C until analysis. The mussels were processed by removing their shells, collecting their soft tissues, which were then homogenized to obtain a uniform mixture. The homogenized tissues were frozen and freeze-dried for ~72 h at −109 °C and <12.8 Pa. Finally, the freeze-dried mussels were ground into a fine powder to increase the extraction efficiency of target analytes. Figure 2 summarizes the sampling and treatment procedures.

### 2.3. Samples Pretreatment

#### 2.3.1. Seawater Pretreatment

Seawater samples were subjected to a filtration step using a glass fiber filter and a nylon membrane filter in order to remove any suspended particle matter. Then, 50 µL of a 50 ng mL^−1^ standard mixture solution was added to 50 mL of the seawater samples for subsequent online solid phase extraction.

#### 2.3.2. Mussels Pretreatment

The mussels were shipped to the laboratory without any manipulation, so the shell had to be separated from the valve for further lyophilization. Once lyophilized, the samples were homogenized, and then 1 g of weight and 5 mL of Ethyl acetate (AcEt) were added to the samples in a centrifuge tube and placed in the ultrasonic bath for 10 min. They were then centrifuged at 8 °C (4000 rpm for 20 min). The obtained supernatant was then transferred to a vial and evaporated using a TurboVap and a stream of nitrogen. They are reconstituted in the vial with 5 mL MeOH and again placed in the ultrasonic bath for 5 min. The extract is vortexed and transferred to a bottle containing 200 mL of H_2_O. The addition of MeOH to the vial is repeated with different volumes of methanol in order to carry the entire amount of sample in the vial. The volumes of MeOH in some samples vary in order to carry the total amount of sample, but generally, a total volume of 8 mL of MeOH was treated. SPE was performed using C18 columns (500 mg and 3 mL). The columns were conditioned with 2 × 2.5 mL AcEt / DCM (Dichloromethane), 2 × 2.5 mL MeOH, and finally 2 × 2.5 mL H_2_O. The sample was injected into the column through capillaries. At the end of the extraction, the column containing the sample was washed with 2 × 2.5 mL of H_2_O to remove possible impurities. The extract was reconstituted again in volumes of less than 1 mL, first with MeOH and then with DCM, to draw the whole sample into a conical vial for further MS/MS analysis. Prior to analysis, the internal standards were added to the sample extract: 100 μL of BZT (benzotriazole), deuterated at 500 ppb, and 100 μL of ISD (internal standards-deuterated) at 500 ppb concentration. The extracts in the vial were then evaporated to dryness in the Reacti-Vap. The residue was finally reconstituted with 500 μL of DCM. It was observed that most of the samples formed a suspension after reconstitution with DCM. The samples were filtered using a 0.2 μm nylon filter. To ensure that as few samples as possible were lost, the filter was conditioned with 1 mL of DCM, and after the filter was washed with 1 mL of DCM to remove any traces of sample that might be trapped in the filter.

### 2.4. Analysis of Seawater Samples

Determination of the chemical oxygen demand (COD), biochemical oxygen demand (BOD_5_), total organic carbon (TOC), absorbable organically bound halogens (AOX), total suspended solids (TSS), and nitrate (NO_3_) was carried out by a Pastel UV-vis (Secomam, Alès, France). The analytical method applied was developed and validated previously [15,22]. To measure the conductivity, we used a WTW 315i conductimeter, turbidity by AQUALITIC^®^ (Dortmund, Germany), and pH with a WTW pH-meter.

### 2.5. Analysis by LC-MS/MS

#### 2.5.1. Seawater Analysis

Filtered seawater samples were subjected to extraction and chromatographic separation using the SymbiosisTM Pico online SPE-LC apparatus (Spark Holland, Emmen, The Netherlands). Online standard and samples were carried out by adding 5 mL matching solutions via a PLRP-s cartridge, which was previously conditioned at a 5 mL min^−1^ flow rate using 1 mL MeOH, 1 mL ACN, and 1 mL HPLC-water. Each cartridge was cleaned with 0.5 mL of HPLC water, after which the chromatography mobile phase eluted the samples onto the LC column. A liquid chromatography column was used for chromatographic separation (Purospher^®^ STAR RP-18 endcapped (2 µm)). The 4000 QTRAP^®^ LC-MS/MS System was used for detection. Analyses were performed using an electrospray ionization probe (ESI+, ESI−). The volume injected was 5 mL. Supplementary Materials includes a list of additional experimental settings in Tables S3 and S4. Gago-Ferrero et al. [23] implemented the performance of the method.

#### 2.5.2. Mussels Analysis

Sample extracts and standards, together with the blank, were analyzed by LC-MS/MS. Elution of the contained solution was carried out via HPLC at a mobile phase, according to the conditions: 0.3 mL per minute. Concerns about positive ionization (PI) mode analyses: the mobile phase A comprises HPLC-grade distilled water and a phase B of MeOH (formic acid at 0.1%). The gradient of elution began with 75% of phase A and 25% of phase B. After 3 min, the aqueous phase was 50% organic. Five minutes after the start of the analysis, the organic phase (B) is present at 100%. Finally, at minute 17, phase A is 75% and phase B is 25%. Two transitions (SRM1 and SRM2) were selected; the more prevalent is utilized for measurement, while the other is used to verify identity. The target PCP ingredients in samples must have a chromatographic retention time within ± 2% of the PCP standards, and the ratio of SRM1/SRM2 cannot deviate from the standard by more than 20–50%. Table S2 describes the LC-MS/MS quality parameters for PCP ingredients analysis. Applied Biosystems/MDS Sciex Symbiosis Pico for Analyst software and Software version 1.4.2 of Analyst were utilized for data collection, instrument control, and evaluation. Recovery rates ranged from 32.7% to 121%, which is typical for multi-residue analytical methods involving compounds with widely varying physicochemical properties, described by Gago-Ferrero et al. [11], García-Gil et al. [24], and Molins-Delgado et al. [25]. Limits of quantitation (LOQs) ranged from 0.1 to 1.0 ng L^−1^, while limits of detection (LODs) ranged from 0.01 to 0.4 ng L^−1^, for the target compounds (Tables S2–S4).

### 2.6. QA/QC in Chemical Analysis

A comprehensive QA/QC protocol was applied, incorporating blanks, matrix-matched standards, isotope-labeled internal standards, and SRM-based compound identification. Recovery rates, LODs, and LOQs were determined to ensure the accuracy, precision, and reproducibility of the results.

The quality control (QC) and quality assurance (QA) standards used included a number of measurements. Wearing gloves throughout the analytical procedure, the use of reagents and solvents specifically dedicated for such processes, and through cleaning of all equipment of the laboratory by using solvents of different polarity (acetone, MeOH, and dichloromethane) as well as additional heating at 350 °C overnight, were among the preventive measures taken to guarantee the consistency of the results. In order to detect probable background levels, analyses were performed on laboratory blanks-pure solvents, and reagents used to prevent memory effects, check for contamination of the instrument, and stop the flow of possibly retained substances. A blank of the method was performed, and quality control samples were introduced randomly in the analysis of the sequence to ensure the quality of the determination. A total of ten newly generated matrix-matched calibration standards were employed to evaluate the linearity and sensitivity over the concentration range of interest. A mixed standard solution (as a control) was included in each analytical session to look for any potential instrumental drift in the response variables. Using the appropriate ^13^C and deuterium-labeled isotope standards. Isotope dilution was used for quantification, which was then assessed as the area of the resulting peaks. The 10 matrix-matched calibration standards and the samples were subjected to the same procedure. All the compounds were identified using the chromatography retention time (RT) and with the two most intense transitions (first to quantify and second to confirm the analyte), as recommended by the European Commission [26].

### 2.7. Estimation of Human Mussel’s Intake

Although there are no studies on how sunscreens affect our bodies through seafood consumption, we can calculate how many pollutants are ingested by eating a portion of mussels. The estimated daily intake (EDI) of a substance is calculated using the following equation from Onsanit et al. [27].

$$EDI=\frac{P\times C}{M}$$

C_PCP ingredients_ = the concentration in each fish sample in mg kg^−1^,

P_food consumption_ = the daily intake of food in kg person^−1^

MW_average_ = the average body weight in kg person^−1^

PCP ingredients in mussel samples, used in the above equation, were the average maximum values of trace element concentrations in the mussel. The consumption rate was 450 g/day, and the body weights for adults were assumed to be 65 kg [28].

### 2.8. Statistical Analysis

The statistical analysis was performed to determine significant differences in the concentrations of personal care product (PCP) ingredients in mussels among different coastal sites and to assess potential correlations between PCP ingredients and the physicochemical parameters of the seawater. Statistical analyses were conducted using R software version 4.2.2 (R Core Team, 2022). One-way ANOVA was used to assess differences in mean concentrations of PCPs in mussels across coastal sites, followed by Tukey’s post hoc test for pairwise comparisons at *p* = 0.05, as recommended for environmental contaminant studies [29,30]. Pearson’s correlation coefficients were calculated to investigate relationships between concentrations of PCP ingredients and physicochemical parameters of the seawater, with significance at *p* < 0.05 [23,31]. These analyses allow evaluation of spatial variation in contamination and potential associations with environmental factors.

## 3. Results

### 3.1. Characterization of Seawater Samples

Table 1 elucidates the physicochemical parameters of the three seawater samples from the Tunisian coastal areas. The results showed that the highest values for conductivity, COD, and TSS (58.51 ± 0.5 mg L^−1^, 24.9 ± 1.8 mg L^−1^, and 48.6 ± 0.4 mg L^−1^, respectively) were found in the seawater from the sampling site of the city of Monastir. As can be seen, the TSS concentrations in seawater in the three sites varied greatly, while most data acquired during the sampling program were kept below permissible limits. In addition, the average level in this study was 37.2 mg L^−1^, exceeding the legal limit of 30.0 mg L^−1^ [32] (N.T. 106.002 (1989)). The elevated total dissolved solids (TSS) in water bodies could result in a drop in dissolved oxygen and a rise in water temperature, which can negatively interfere with aquatic life. Furthermore, the increased TSS is typically associated with elevated levels of metals, minerals, bacteria, and organic contaminants in seawater [18].

Correspondingly, the pH of all samples analyzed remained close to neutral, ranging from 7.01 ± 0.1 to 7.67 ± 0.3 mg L^−1^. Furthermore, the biodegradability potential of the organic matter in the samples was estimated based on the COD/BOD_5_ ratio. Our study showed average ratios between 2.24 ± 0.2 and 2.8 ± 0.2. Therefore, a potential biodegradation should be expected. It is important to consider that nitrates and nitrites are recognized to be hazardous compounds for aquatic biota. The concentrations of AOX and NO_3_ in all effluent samples were less than 0.5 mg L^−1^, which is within the permissible limits of detection.

### 3.2. Occurrence of Personal Care Product Ingredients

#### 3.2.1. Seawater

Table 2 shows the concentration of the target PCP ingredients in each of the seawater samples reported in the present study. Thirteen of the 18 personal care product ingredients analyzed were detected. AVO was found in all seawater samples in a range between 524.94 ± 2 and 621 ± 3.9 ng L^−1^. The maximal concentration was encountered in Mahdia, Sousse, and Monastir. Thus, the BP3 occurrence in seawater was detected at low levels in all samples (ranging from 18.76 ± 1.3 to 29.01 ± 1 ng L^−1^). 4HB was also significantly detected at the highest level in seawater from Sousse (139.75 ± 1.9 ng L^−1^). As for BP1, UV328, and BP2, they were detected at similar contents in the different samples studied. Nevertheless, the sampling sites exhibited no significant differences. The benzotriazole found in the present work was 5,6-Dimethyl-1H-benzotriazole (DMBZT), mainly in Monastir (35.073 ± 0.7 ng L^−1^). The concentration ranges for the most common PCP ingredients in seawater from 3 Tunisian beaches are summarized in Table 2. As illustrated, a number of factors, such as the distance between the sampling sites and the discharge points of the sewage treatment plants and the morphology of the sea (open or semi-enclosed), influence the concentrations of these organic UV filters detected, which vary greatly depending on the sampling site. UV filters typically have high boiling temperatures (400 or higher) and are made of non-volatile materials. With the exception of a few benzophenone derivatives, their medium to high (log KOW > 3) and relatively low aqueous solubility is considered hydrophilic. This is likely to lead to bioaccumulation in sediments and marine biota following discharge into coastal seas.

#### 3.2.2. Mussels

**Figure 3:**
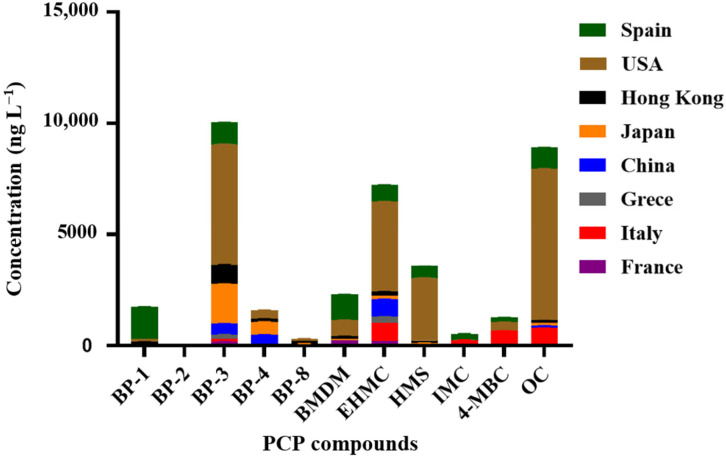
The occurrence of PCP ingredients in seawater all over the world. Data are from [33,34,35,36,37,38].

Table 3 illustrates the concentration values found for the UV filters studied. Thirteen of the 18 compounds analyzed were detected. All samples analyzed were contaminated with at least two compounds. The concentration of the pollutants is expressed in ng g^-1^. As can be seen, some contaminants are more abundant than others. The sunscreen 4HB is present in almost all samples. Secondly, there is the pollutant BP1, which is present in all countries. We can say that in the sunscreen family, benzophenones are the most abundant. Therefore, BP2 was identified as the parent chemical and seemed to be less susceptible to human metabolism because it is less metabolized. The mean maximum concentration for BP2 was 7.103 ± 0.2 ng g^−1^ in Mahdia. The benzotriazoles are the least abundant sunscreens in the samples because TBHPBT, UV328, and DMBZT are less abundant contaminants. In our study, the concentrations of AVO were particularly high, with a maximum value of 193.481 ± 5.5 ng g^−1^ in the city of Sousse. 4HB is one of the metabolites of UVFs of the benzophenone class. The median concentrations of 4HB were exceptionally high, ranging from 24.645 ± 0.5 ng g^−1^ to 26.745 ± 0.4 ng g^−1^ of mussels in Monastir and Sousse, respectively. Interestingly, the concentrations observed for the benzotriazole family corresponded to 2-(5-t-butyl-2-hydroxyphenyl) benzotriazole (TBHPBT), with a maximum of 26.704 ± 1.7 ng g^−1^ of mussels in Sousse (Figure 3).

## 4. Discussion

In the current study, the selection of standards employed in the evaluation process is informed by two key sources: local market surveys and preliminary screenings. Some compounds that are currently banned or restricted in the European Union, such as 3-(4-methylbenzylidene camphor) (4-MBC), are still permitted and used in products available on the Tunisian market, justifying their incorporation in the study. Furthermore, from a toxicological standpoint, the persistence of certain banned compounds in the environment, in conjunction with their potential for bioaccumulation, renders them relevant targets within the domain of pollution studies. 4-MBC, for instance, has been demonstrated to possess endocrine-disrupting properties and has previously been detected in aquatic environments with measurable impacts on marine organisms.

Very few studies have found personal care product ingredients in seawater and mussel samples. Accordingly, we have examined the occurrence of benzotriazole and benzophenone-type UV filters by LC-MS/MS. It is worth noting the presence of BP2 in relation to the UV filters in the analyzed mussels and seawater. The structural symmetry and small size of these compounds allow them to penetrate tissues, which is why it is known to bio-concentrate in aquatic biota [1]. Our results showed that the two major compounds were AVO and TBHPBT (both in seawater and mussel samples). Accordingly, the lowest Kow for avobenzone (AVO) and TBHPBT, and its higher solubility, contribute to the high concentrations in seawater and mussels. In fact, we speculate that the higher kinetics of AVO and TBHPBT degradation occurring in the seawater, where photolysis is probably stronger, were the cause of this fractionation. The two other organic UV filters, on the other hand, did not have this effect because they were less water-repellent and had longer half-lives, which caused too much of them to be in the samples [39]. The pollutant BP3 is metabolized by the organisms to form its metabolites, BP1, 4HB, and 4DHB. Therefore, this may be one reason why we find BP3 in the samples, while the pollutants 4HB and BP1 are the most abundant. BP3 is a molecule that is found in personal healing product ingredients around the world (Figure 3), but at a maximum permissible concentration. Table S5 illustrates the chemical properties of the investigated compounds. Currently, the exposure to Xenobiotic compounds, which are capable of disrupting or modulating the endocrine system, has become a real threat. In recent years, many reports on the absorption through the dermal route of BP1 (similar in structure to estrogens) have shown that it can pass over the skin and enter the body. Therefore, according to the European Commission Science for Environment Policy, we are exposed to a harmful compound for health found in everyday products such as PCP ingredients [40]. In the current work, the high levels can be explained by the octanol–water partition coefficient (logKow 3.17), which indicates that BP1 is a lipophilic molecule with a tendency to bioaccumulate in fish, shellfish, and other tissues by adsorption to suspended matter and sediment [1,23].

The average levels of 4HB, one of the major metabolites of UV benzophenone type, were particularly high. This finding is of concern because 4HB was reported to cause significant DNA alteration and genotoxicity that are considerably worse than those of the parent molecule [41]. Beyond the scope of this study and for future research, the report “State of the Climate in Europe in 2022” was jointly created by the European Union’s Copernicus Climate Change Service (C3S) and the World Meteorological Organization (WMO). It demonstrated that in 2022, surface solar radiation hit record highs since records first started in 1983, exceeding the 1991–2020 average by 4.9%. In particular, Tunisia has an UV index of 10, which indicates the alarming situation of sun-related skin damage from ultraviolet radiation. As a result, UV protection solutions are expected to become more popular.

In our study, the high concentrations of the benzotriazole family observed in the samples are probably due to hospitalization use of drugs made up of BZT derivatives. In fact, BZT is indeed present in HWWs, which is explained by its wide range of clinical uses in new-generation medications, including anticancer medications as well as antioxidant, antifungal, antibacterial, antiviral, antitubercular, anthelmintic compounds, and antihypertensive [19]. As a result, ecotoxicity data and environmental monitoring may be used to design suitable laws. Once administered, these compounds metabolize, degrade, and ultimately release the BZT moiety into seawater and eventually into molluscs (mainly *Mytilus galloprovincialis*). Once released, benzotriazoles persist because they are highly soluble and poorly biodegradable (long half-life ranging from one month to at least one year) [3].

**Figure 4:**
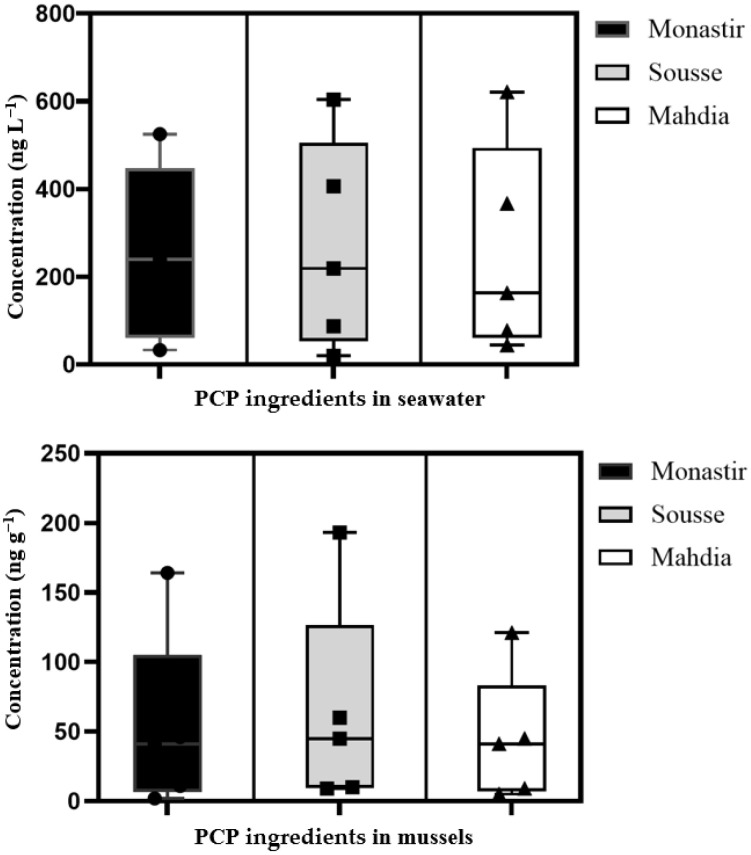
Distribution of personal care product ingredients in coastal waters and mussels from Monastir, Sousse, and Mahdia in Tunisia.

As far as we know, there has never been a direct correlation between BZT residues and clinical medications. This significant discovery highlights the significance and alarming source of BZT pollution in the ecosystem: untreated hospital wastewater. Usually combined with municipal wastewater, these effluents are treated, but are not effectively eliminated during the treatment process [25], leading to their eventual release into the environment. Due to their high solubility and short half-lives of days in the environment, PCP ingredients persist in the aquatic environment [42]. Generally, the reported total concentrations of UVFs in North Africa are at least one to two times more massive than those in Europe and Asia; therefore, the detection frequencies are significantly higher, as shown by a comparison of the prevalence of UVFs in aquatic organisms in Europe and Asia. The main reason for this observation is most likely the difference in the usage of skin hygiene products between the areas of the study. In North Africa, sunscreen use is reported to be much higher than in Europe and Asia (due to the higher average annual temperature). From these results, it was possible to calculate the total pollutant concentration per city and also its average. The results showed that, according to the mussel samples analysis, Sousse is by far the most polluted city. Monastir follows in second place, but at a much lower level. The third most contaminated city is Mahdia. Mussels are, therefore, considered a biomarker for pollution by personal care product ingredients in coastal areas (Figure 4). Also for coastal monitoring and water quality monitoring in the Tunisian Sahel.

In the recent decade, PCP ingredients have been reported worldwide: waters from rivers and lake sediments and from Asia and Europe [43,44,45]; sediments, seawater, sand from North America, Asia, and the Mediterranean region [46,47,48]. In this work, the observed occurrence may be partly related to a massive tourist destination [13,49]. The Tunisian Sahel is situated in the Tunisian central east and comprises three governorates, Monastir, Mahdia, and Sousse, which are considered to be the perfect holiday destination for tourists. Millions of tourists visit the Sahel every year, mainly between April and October. To assemble a few details regarding seasonality in the coastal area of Tunisia, samples were selected during the spring and summer periods. Every sample in this season included at least seven PCP ingredients investigated in this study. The occurrence of PCP ingredients was strongly influenced by summer and spring because of their relatively high concentration in this period [50]. Several studies have identified daily and seasonal variations as the primary causes [46,49,51]. In fact, both are correlated with the number of visitors (9.4 million arrivals annually). On the other hand, the concentrations of UV filters also varied spatially, and in most marine environments became less concentrated as one moved away from the coastline to the beach and away from the source of emissions. The accumulation of sunscreen ingredients is also influenced by the water circulation pattern in each bay, beach, and coastal area. In addition to the aforementioned spatial variations in UV filter concentrations in coastal areas, there are also temporal fluctuations at different scales. The distribution of certain UV filters in the surface microlayer of the Spanish island and Mallorca during the course of a day has been described by Tovar-Sánchez et al. [46]. The highest levels were found at midday, coinciding with the peak of solar radiation. Positive correlations between water concentrations and population density and diurnal variations have been found in other locations heavily impacted by recreational activities [52]. On a longer time scale, warmer months are more likely to have higher UV filter concentrations than colder months. For example, silicon-based passive samplers were used to determine the time-weighted average concentrations of specific organic UV filters in surface waters of the Bay of Cadiz (Spain) in 2015. There was a notable seasonal pattern linked to changes in the population due to the abundance of tourists, which peaks in the summer. The highest concentrations (up to 500 ng L^−1^) were recorded from June to September [51].

The coasts of Monastir, Sousse, and Mahdia showed the highest abundance in the summer season in Tunisia [53]. This explains the reason that the samples contain a higher load of PCP ingredients in this area. As described, this region is close to wastewater treatment plants and affected by the unlawful disposal of hazardous effluents, which have the effect of aggravating the environmental risk. In addition, PCP ingredients revealed higher concentrations in the spring and summer seasons (March to August), since consumption grew during this season, which explained that the PPCs contamination in the seawater was related to the summer tourism and dense population. Our findings are in line with those of previous works, which reported that the massive usage of sunscreens and the increase in tourists who came during the summer period led to an increase in the concentrations of PCP ingredients in the marine environment [54]. For example, the number of beachgoers has been correlated with UV filters in beach waters. Tovar-Sánchez et al. [46] have described the occurrence of (benzophenone-3 (BZ-3) and 4-methylbenzylidene camphor (4-MBC) in the surface microlayer during one day around the Island of Majorca (Spain). In fact, they have found a direct link between beachgoers and PCP ingredients: the concentration of UV filters in the surface water increased, with the number of visitors during the warm season. Like Picot-Groz et al. [49], they also carried out the daily fluctuation of UV filters in mussel samples. For example, EHMC has been found in increased amounts in crustaceans, fish, and bivalves [17]. Our results support the idea that PCP ingredients can bioaccumulate in coastal matrices. Several studies have suggested their bioaccumulation in marine biota. Rodil et al. [55] evaluated the presence of UV Filters, including OC- (Octocrylene), (0–141 ng g^−1^, EMC (0–94 ng g^−1^), BP3 (0–63 ng g^−1^), and OD-PABA (octyldimethyl para-aminobenzoic acid) (0–12 ng g^−1^), and 4-MBC (0–49 ng g^−1^), in bivalves (Cerastoderma edule, *Mytilus galloprovincialis*, and *Ruditapes decussatus*), and they were much higher than those in our study. As mentioned above, Vidal-Liñán et al. [6] demonstrated that *Mytilus galloprovincialis* accumulated 4-MBC, BP3, BP4, OC, and OD–PABA at concentrations 801, 67, 520, 559, and 13.5 ng g^−1^, respectively. This means that personal care product ingredients can bioaccumulate in aquatic organisms and are capable of accumulating along the food chain. Even very low concentrations of these pollutants in water or sediment can become toxic at higher levels of the food chain [56]. In general, the ratio between the concentration of a chemical and its environment is referred to as the bioaccumulation factor (BAF). According to the European Chemicals Agency (ECHA), critical BCF thresholds agreed for aquatic ecosystems are 2.000 and 5.000 [57]. By considering, UV filters employed in personal care product ingredients generally exhibited BAF levels absolutely permissible value of 2.000 [7]. Interestingly, bioaccumulation may be the best indicator of the presence of PCP ingredients in seawater. The novelty of this study is important because it contributes, for the first time, to a better understanding of the risk posed by PCP ingredients to marine organisms in the Tunisian sea.

The key reason for the accumulation of UVFs in *Mytilus galloprovincialis* is that these latter are characterized by chemical compounds with poor solubility, associated with an elevated octanol-water partition coefficient (Log Kow) [58,59]. In fact, the higher hydrophobicity of a chemical increases its ability to bioaccumulate in marine organisms [60]. It should be borne in mind that discharges to aquatic environments are generally mixed with domestic effluent and treated in specific plants, whereby they are not efficiently removed [25] and thus released to the aquatic medium. For instance, benzotriazoles accumulated in the environment due to their extended half-lives and recalcitrance [61,62].

The detrimental effects of exposure to BPs, MBCs, and PABAs on living organisms in aquatic ecosystems have been the subject of much attention. When we use products containing UV filters, they penetrate the skin’s layers and can interfere with the endocrine system. As they penetrate, they can cause adverse effects such as atopic dermatitis or mutagenic effects. Once they have passed through the skin layers, they can modulate or disrupt the endocrine system because the structure of 2-Hydroxy-4-methoxybenzophenone (BP3) and 2-ethylhexyl trans-4-methoxy cinnamate (EHMC) is similar to that of estrogens. Shellfish intake is another way by which pollutants can reach our bodies. It would also be important to consider how these pollutants affect our bodies through the consumption of seafood, but no studies have been reported so far. However, numerous works have demonstrated the presence of selected UV filters in biological fluids and tissues such as the placenta. In this context, several reports have established the deleterious effects of UV-filters, which were observed in bivalves and algae, such as mortality, polyp retraction [63,64], DNA damage [65], morphological abnormalities and behavioral effects have been demonstrated in fish [66,67].

The comparison of accumulated total suspended solids (TSS) in seawater and the occurrence of PCP ingredients in mussels showed that TSS and UV328 were intensely correlated (0.93). AVO, BP1, and 4HB groups also showed a positive correlation (0.67, 0.78, and 0.82, respectively) (Table S6). PCP ingredients in seawater and mussels may be a sign that WWTPs are unable to adequately retain large amounts of particle matter and discard micropollutants of chemical nature from effluent before its discharge into the aquatic environment. Due to the numerous challenges associated with sampling and treatment, only limited investigations have focused on the adsorption of PCP ingredients on suspended matter in effluent [18,19]. Such a fact could explain the augmented levels of some compounds (UV328, ODPABA, AVO, 4HB, BP1), probably due to the higher concentration of PCP ingredients in hospital effluents [19] in Monastir, Mahdia, and Sousse cities (which are very close to the sea). In addition, PCP ingredients adsorbed on suspended solids that later settle in seawater show a higher accumulation rate in marine organisms.

With the aim to determine the estimated daily intake of a substance, the EDI was calculated to be approximately 68.36 (ng kg body-weight^−1^ day^−1^). A portion of mussels weighs about 450 g, but this weight also includes the shell of the mussel. The actual amount eaten is 338 g, as 25% of the weight of the mussel is the shell. Therefore, when we are consuming mussels, we ingest approximately 91,209.3 ng UVFs per portion. The EDI values calculated for this group of emerging pollutants were compared with those evaluated for certain persistent organic contaminants (POPs) in fish. The compounds hexabromocyclododecane (HBCD) and perfluorooctanosulfonic acid (PFOS) reached values of 2.58 (ng kg body-weight^−1^ day^−1^) and 2.7 (ng kg body-weight^−1^ day^−1^), respectively. These contaminants are regulated and have different adverse biological effects. The pollutants studied in this work also have negative biological effects, but are not regulated. As we can see, the concentration of UV filters we ingest (9.88 (ng kg body-weight^−1^ day^−1^) is much higher than that of HBCD and PFOS compounds. The high concentrations of UVFs in the mussels in this work could be a safety concern, given the endocrine-disrupting effects of these molecules and the absence of appropriate information regarding their risk to human health. Nevertheless, a list of personal care product ingredients has been identified as potentially deleterious to marine organisms and should be taken into consideration in any coastal monitoring programs in the future.

While this study provides novel insights into the occurrence and bioaccumulation of personal care product (PCP) ingredients and UVFs in seawater and mussels from the Tunisian Sahel, certain limitations should be acknowledged. The sampling was limited to three coastal cities over a six-month period, primarily during the spring and summer months. This may not fully reflect year-round variability or conditions in other regions. The analysis was conducted on a selection of UVFs and benzotriazole compounds, which may have resulted in an underestimation of the total chemical burden present in the marine environment. Analytical challenges, including matrix effects in LC-MS/MS, may have influenced measured concentrations, and the estimated daily intake (EDI) for human exposure does not capture cumulative or long-term effects. Moreover, while positive correlations were observed between PCP ingredient concentrations, total suspended solids, and proximity to wastewater sources, causality cannot be firmly established. It is evident that the study did not thoroughly explore the degradation pathways or the formation of unknown metabolites, which could limit the understanding of the long-term environmental fate and toxicity. Despite these limitations, the findings underscore significant ecological and potential human health risks, thereby providing a strong foundation for future monitoring and risk assessment of PCP ingredients in coastal marine environments.

## 5. Conclusions

Our results demonstrated the presence of both personal care product (PCP) ingredients and UV filters in seawater and in *Mytilus galloprovincialis* from the studied Tunisian coastal areas, confirming that mussels can serve as an effective biomarker for monitoring contamination with these compounds.

The examination of mussel samples from different origins of coastal areas in Tunisia revealed that the biota samples accumulated UVF residues. The most abundant UV filters in marine biota were those belonging to the families of benzophenones, benzotriazoles, and parabens, in particular, the compound avobenzone. This means that the organisms have some kind of detoxification mechanism by which the UVFs are transformed to be excreted. However, the metabolites produced were also found to be accumulated by the organism. Moreover, the amount of emerging pollutants ingested by a Tunisian person in a portion of Tunisia mussels is 91,209.3 ng UVFs. Our results showed that the situation on the Tunisian Sahel coast is alarming. Hence, to address this issue, rigorous monitoring programs in Tunisian coastal areas are of priority. As more research is needed to understand these compounds’ mobility, bioavailability, and toxicity, the true scope and possible implications of their intake into marine ecosystems remain unknown. In addition, expanding the study to encompass more popular cosmetic ingredients, including octocrylene, has the potential to enhance the reliability and consistency of the present work. Finally, in order to ensure the sustainable development of coastal communities and raise public awareness of the environmental effects of PCP ingredients and UVFs in the marine ecosystem, it is imperative that this issue be approached from three different angles: social, scientific, and regulatory.

## Figures and Tables

**Table 1:** Results of the physicochemical analysis of seawater samples.

Governorates Parameters	Monastir	Sousse	Mahdia
BOD_5_ (mgO_2_ L^−1^)	9.3 ± 0.3	8.28 ± 0.7	8.5 ± 0.6
COD (mgO_2_ L^−1^)	24.9 ± 1.8	23.24 ± 1.2	19.60 ± 0.2
TSS (mg L^−1^)	48.6 ± 0.4	37.5 ± 0.5	25.55 ± 0.3
TOC (mg L^−1^)	2.62 ± 0.5	3.05 ± 0.2	2.56 ± 5.6
NO_3_ (mg L^−1^)	<0.5	<0.5	<0.5
AOX (mg L^−1^)	<0.5	<0.5	<0.5
Turbidity (NTU)	0.81 ± 0.1	0.73 ± 0.5	0.3.6 ± 0.2
Conductivity (ms cm^−1^)	58.51 ± 0.5	55.15 ± 1	35.69 ± 1
pH	7.09 ± 0.2	7.67 ± 0.3	7.01 ± 0.1
COD/BOD_5_	2.69 ± 0.6	2.8 ± 0.2	2.24 ± 0.2
OM (mg L^−1^)	2.4 ± 0.8	3.33 ± 0.8	1.66 ± 0.7

BOD_5_: biochemical oxygen demand; COD: chemical oxygen demand; TSS: total suspended solids; TOC: total organic carbon; NO_3_: nitrates; AOX: absorbable organically bound halogens; COD/BOD_5_: ratio of the biodegradability; OM: Organic materials.

**Table 2:** Concentration of the target compounds (PCP ingredients) in the seawater samples (ng L^−1^). Values with different letters indicate significant differences according to the Tukey test (*p* < 0.05).

PCP Ingredients	Seawater			LOD (ng L^−1^)	LOQ (ng L^−1^)
City	Monastir	Sousse	Mahdia		
BP1	82.23 ± 0.8 ^b^	91.98 ± 2.4 ^a^	89.46 ± 2.3 ^a^	1.09	3.56
BP2	62.86 ± 2.7 ^a^	74.51 ± 3.9 ^b^	71.03 ± 2.7 ^b^	1.17	3.90
BP3	25.27 ± 1 ^b^	29.01 ± 1 ^a^	18.76 ± 1.3 ^b^	0.74	2.47
4HB	132, 45 ± 1.5 ^a^	139.75 ± 1.9 ^a^	125. 61 ± 2.2 ^ab^	1.04	3.47
4DHB	67. 57 ± 2.8 ^a^	71.05 ± 1.8 ^a^	61. 85 ± 2.7 ^b^	0.99	3.30
AVO	524.94 ± 2 ^a^	603.70 ± 4.4 ^a^	621.02 ±3.9 ^b^	0.93	3.75
EtPABA	51.72 ± 2.9 ^b^	41.89 ± 1.4 ^ab^	40.94 ± 1.2 ^a^	0.3	1.1
ODPABA	36.72 ± 0.8 ^a^	45.27 ± 1.4 ^a^	35.83 ± 0.4 ^a^	0.73	2.44
EHMC	32.64 ± 0.9 ^a^	19 ± 0.7 ^c^	43.87 ± 0.7 ^b^	1.71	5.69
TBHPBT	83.42 ± 1.2 ^a^	74.27 ± 1.3 ^a^	62.77 ± 0.8 ^b^	0.1	0.3
UVP	59.72 ± 0.9 ^a^	66.97 ± 3.4 ^b^	19.43 ± 0.2 ^c^	1.03	3.42
UV328	60.47 ± 1 ^b^	58.27 ± 2.4 ^a^	58.01 ± 2.7 ^a^	1.03	3.42
DMBZT	35.073 ± 0.7 ^b^	19.29 ± 0.9 ^a^	22.46 ± 0.8 ^b^	0.90	2.99

Different letters over the bars indicate significant differences among treatments according to Tukey’s test (*p* < 0.05).

**Table 3:** Concentration of the target compounds (PCPS) in the mussel samples (ng/g). Not detected (n.d.). Values with different letters indicate significant differences according to the Tukey test (*p* < 0.05).

PCP Ingredients	Mussels			LOD (ng g^−1^)	LOQ (ng g^−1^)
City	Monastir	Sousse	Mahdia		
BP1	12.53 ± 0.5 ^b^	7.216 ± 0.4 ^a^	8.246 ± 0.2 ^a^	1.09	3.56
BP2	4.360 ± 0.1 ^a^	4.503 ± 0.1 ^a^	7.103 ± 0.2 ^ab^	1.17	3.90
BP3	n.d.	0.391 ± 0.1 ^a^	n.d.	0.74	2.47
4HB	24.645 ± 0.5 ^a^	26.745 ± 0.4 ^a^	25.465 ± 0.3 ^ab^	1.04	3.47
4DHB	6.652 ± 1.5 ^a^	7.85 ± 2.7 ^a^	1.025 ± 0.5 ^b^	0.99	3.30
AVO	164.244 ± 2.8 ^a^	193.481 ± 5.5 ^a^	121.076 ± 1.6 ^b^	0.93	3.75
EtPABA	5.553 ± 1 ^b^	4.030 ± 0.9 ^ab^	3.091 ± 0.7 ^a^	0.3	1.1
ODPABA	6.612 ± 0.26 ^a^	5.96 ± 1.79 ^a^	5.553 ± 0.7 ^a^	0.73	2.44
EHMC	2.316 ± 0.8 ^a^	9 ± 1.43 ^c^	4.360 ± 0.8 ^b^	1.71	5.69
TBHPBT	23.886 ± 2.2 ^a^	26.704 ± 1.7 ^a^	20.987 ± 0.7 ^b^	0.1	0.3
UVP	9.652 ± 0.7 ^a^	16.566 ± 1.9 ^ab^	8.043 ± 0.8 ^b^	1.03	3.42
UV328	5.224 ± 1.46 ^b^	14.747 ± 3.01 ^a^	13.045 ± 1.9 ^a^	1.03	3.42
DMBZT	4.503 ± 0.5 ^b^	1.224 ± 0.5 ^a^	2.96 ± 0.6 ^b^	0.90	2.99

Different letters over the bars indicate significant differences among treatments according to Tukey’s test (*p* < 0.05).

## Data Availability

All data and materials are available in the Supplementary Materials.

## Supplementary Materials

The following supporting information can be downloaded at: https://www.mdpi.com/article/10.3390/toxics13100847/s1, Table S1: Chemical standards analyzed in the present study with their corresponding INCI names and function in cosmetic products [68,69,70,71,72,73,74,75,76,77,78]; Table S2: Instrumental quality parameters of the HPLC- MS/MS method for the analysis of PCP ingredients; Table S3: Gradient, mobile phases and flow used in positive ionization; Table S4: Gradient, mobile phases and flow used in negative ionization; Table S5: Chemical properties of the investigated compounds; Table S6: Pearson correlation values between concentrations of TSS and PCP ingredients in samples.
