# Supplementary material for: Mytilus galloprovincialis as a Biomarker for Personal Care Product (PCP) Ingredients and UV Filters (UVFs) in Tunisian Coastal Waters: Correlation with the Chemical Composition of Polluted Seawater
Source: Toxics. 2025 Oct 6;13(10):847. doi: 10.3390/toxics13100847 (PMC12567776; doi:10.3390/toxics13100847)
Supplement: Supplementary file 1 [file toxics-13-00847-s001.zip › toxics-3879960-supplementary.pdf]

Supplementary Materials

Mytilus galloprovincialis as a Biomarker for Personal Care Product (PCP) Ingredients and UV Filters (UVFs) in Tunisian Coastal Waters: Correlation with the Chemical Composition of Polluted Seawater

Emna Nasri [1,2], Elhem Bouchiba [1], Bouthaina Brahmi [1], Siwar Bouyahi [1], Eduardo Alberto López-Maldonado [3,*] and Mohamed Ali Borgi [1,*]

[1] Laboratory of Biotechnology and Biomonitoring of the Environment and Oasis Ecosystems (LBBEEO), Faculty of Sciences of Gafsa, University of Gafsa, University Campus of Zarroug, Gafsa 2112, Tunisia; emmna.nasri@gmail.com (E.N.); ebouchiba482@gmail.com (E.B.); brahmi01@yahoo.com (B.B.); bouyahisiwar8@gmail.com (S.B.)
[2] Faculty of Sciences of Tunis, University of Tunis El Manar, 1068 Tunis, Tunisia
[3] Faculty of Chemical Sciences and Engineering, Autonomous University of Baja California, Tijuana 22424, Baja California, Mexico.
* Correspondence: elopez92@uabc.edu.mx (E.A.L.-M.); borgima@fsgf.ugaf.tn (M.A.B.)

Table S1. Chemical standards analyzed in the present study with their corresponding INCI names and function in cosmetic products.

Abbreviation	Chemical Name	INCI Name	Function in Cosmetic Products	Reference
BP3	Benzophenone-3	Oxybenzone	UV filter (sunscreen agent)	[68–72]
BP1	Benzophenone-1	Benzophenone-1	UV absorber, stabilizer in formulations	[68,69,73]
BP2	Benzophenone-2	Benzophenone-2	UV absorber, photostabilizer	[68,69,73]
BP4	Benzophenone-4	Benzophenone-4	UV filter (water-soluble), sunscreen agent	[68,69,73]
4HB	4-Hydroxybenzoic acid	4-Hydroxybenzoic Acid	Preservative intermediate, antimicrobial function	[68,69]
4DHB	4,4′-Dihydroxybenzophenone	Dihydroxybenzophenone	UV absorber, stabilizer	[68,69]
DHMB	2,4-Dihydroxybenzophenone	Dihydroxybenzophenone-2	UV absorber, photostabilizer	[68,69]
AVO	Butyl Methoxydibenzoylmethane	Avobenzone	UV filter (broad spectrum)	[68,69,71,72,74]
4MBC	4-Methylbenzylidene Camphor	Enzacamene	UV filter	[68,69,71,72,75]
EHMC	Ethylhexyl Methoxycinnamate	Octinoxate	UV filter	[68,69,71,72,74]
Et-PABA	Ethyl 4-aminobenzoate	Ethyl PABA	UV filter (less used today)	[68,69,76]
BZT	Benzotriazole	Benzotriazole	UV absorber, stabilizer in cosmetics and plastics	[69,72,73]
MeBZT	Methylbenzotriazole	Methylbenzotriazole	UV absorber	[69,73]
DMBZT	Dimethylbenzotriazole	Dimethylbenzotriazole	UV absorber	[69,73]
UVP	2-Phenylbenzimidazole-5-sulfonic acid	Ensulizole	UV filter (water-soluble)	[68,69,77]
BePB	Benzylparaben	Benzylparaben	Preservative (antimicrobial)	[68,69,78]
BuPB	Butylparaben	Butylparaben	Preservative (antimicrobial)	[68,69,78]
PrPB	Propylparaben	Propylparaben	Preservative (antimicrobial)	[68,69,78]
MePB	Methylparaben	Methylparaben	Preservative (antimicrobial)	[68,69,78]

Table S2. Instrumental quality parameters of the HPLC- MS/MS method for the analysis of PCP ingredients.

Compound	Linearity Range (μg L^{-1})	R2	RSD % Intra-Day	RSD %Inter-Day	Recovery (%)	RSD (%) n = 2
Benzophenone 1 (BP1)	1-500	0.9989	6	10	58.5	7.1
Benzophenone 2 (BP2)	1-500	0.9994	-	-	45.5	14.3
Benzophenone 3 (BP3)	1-500	0.9996	1	4	65.9	5.6
4-hydroxybenzophenone (4HB)	1-500	0.9992	2	3	44.1	4.9
4,4'-dihydroxybenzophenone (4DHB)	1-500	0.9988	1	4	63.4	3.5
2,2'-dihydroxy-4-methoxybenzophenone (DHMB)	1-500	0.9988	-	-	60.3	8.4
Avobenzone (AVO)	1-500	0.9996	-	-	71.3	1.5
3-(4-Methylbenzylidene)camphor (4-MBC)	1-500	09994	1	6	77.9	1.2
Ethyl-4-p-aminobenzoic acid (EtPABA)	1-500	0.9986	7	8	78.8	3.5
Ethylhexyl dimethyl-4-p-aminobenzoic acid (ODPABA)	1-500	10.000	3	6	75.6	3.3
2-ethylhexyl trans-4-methoxycinnamate (EHMC)	1-500	0.9939	-	-	8.28	3.3
2-(5-tert-butyl-2- hydroxyphenyl)- benzotriazole (TBHPBT)	1-500	0.9991	5	7	71.4	12.1
2-(2H-Benzotriazol-2-yl)-p-cresol (UVP)	1-500	0.9963	4	13	70.4	2
2-(2H-benzotriazol-2-yl)-4,6-biz(1-methyl-1-phenylethyl)pheno (UV234)	1-500	0.9994	2	3	-	-
2-(3,5-di-tert-butyl-2-hydroxy-phenyl)-5-(chlorobenzotriazole (UV327)	1-500	0.99946	3	4	-	-
2-(3,5-di-tert-amyl-2-hydroxyphenyl)benzotriazole (UV328)	1-500	0.9989	1	4	79	9.4
2-(2-hydroxy-5-tert-octylphenyl)-Benzotriazole (UV329)	1-500	0.9979	1	4	-	.
5-Methyl-(1H-Benzotriazol) (MeBZT)	1-500	0.9996	3	9	71.6	12.1
5,6-Dimethyl-1H-benzotriazole (DMBZT)	1-500	0.9993	-	-	-	-

Table S3. Gradient, mobile phases and flow used in positive ionization.

Time (min)	Flow	Positive Ionization	
		% A (MeOH 0.1% F.A.)	%B (H_2O 0.1% F.A.)
0	0.3 mL min^{-1}	5	95
7	0.3 mL min^{-1}	75	25
10	0.3 mL min^{-1}	100	0
17	0.3 mL min^{-1}	100	0
18	0.3 mL min^{-1}	5	95
23	0.3 mL min-^{1}	5	95

F.A Formic Acid

Table S4. Gradient, mobile phases and flow used in negative ionization.

Time (min)	Flow	Positive Ionization	
		% A (MeOH 5 Mm Ac.NH4)	%B (H2O 5 Mm Ac.NH4)
0	0.3 mL min^{-1}	5	95
3	0.3 mL min^{-1}	50	50
6	0.3 mL min^{-1}	90	10
13	0.3 mL min^{-1}	100	0
18	0.3 mL min^{-1}	5	95
20	0.3 mL min^{-1}	5	95

Ac.NH4 Ammonium acetate

Table S5. Chemical properties of the investigated compounds.

Acronym	Name	CAS (Chemical Abstracts Service) Number	Log Kow	Solubility
**BP1**	2,4- dihydroxybenzoph enone	131-56-6	3.17	413.4 mg L^{-1}
**BP2**	2,2′,4,4′- tetrahydroxybenzo phenone	131-55-5	2.78	398.5 mg L^{-1}
**BP3**	2-hydroxy-4- methoxybenzophe none	131-57-7	3.79	140 mg L^{-1}
**4HB**	4- hydroxybenzophe none	1137-42-4	3.02	410 mg L^{-1}
**4DHB**	4,4′- dihydroxybenzoph enone	611-99-4	2.55	45 mg L^{-1}
**DHMB**	2,2′-dihydroxy-4- methoxy- Benzophenone	131-53-3	3.82	27 mg L^{-1}
**AVO**	Avobenzone	70356-09-1	4.51	1517 mg L^{-1}
**EHMC**	2-ethylhexyl trans- 4-methoxy cinnamate	83834-59-7	5.8	0.42 mg L^{-1}
**4MBC**	3-(4-methylbenzylidene)camphor	36861-47-9	4.95	0.1-5.1 g L^{-1}
**OD-PABA**	2-ethylhexyl 4- (dimethyl- amino)benzoate	21245-02-3	6.15	0.6963 mg L^{-1}
**EtPABA**	Ethyl 4- aminobenzoate	94-09-7	1.86	60.1 mg L^{-1}
**BZT**	1H-Benzotriazol	2170-39-0	5.3	28 mg L^{-1}
**MeBZT**	5-Methyl-(1H-benzotriazol)	136-85-6	1.89	70 mg L^{-1}
**DMBZT**	5,6-Dimethyl-1H-benzotriazole	131-53-7	2.06	914 mg L^{-1}
**UVP**	2-(2H-Benzotriazol-2-yl)-p-cresol	2440-22-4	4.3	25,95 mg L^{-1}

Table S6. Pearson correlation values between concentrations of TSS and PCP ingredients in samples.

Correlation Values	TSS	BP1	BP2	BP3	4HB	4DHB	AVO	EtPABA	ODPABA	EHMC	TBHPBT	UVP	UV328
**TSS**	1.00	**0.78**	0.08	-0.01	0.63	-0.27	0.43	-0.31	-0.42	0.05	0.63	0.44	**0.93**
**BP1**	**0.78**	1.00	0.50	0.10	0.63	0.03	0.73	0.38	0.04	0.19	0.08	0.21	0.19
**BP2**	0.08	0.50	1.00	0.09	0.43	0.06	0.32	0.12	0.06	0.09	0.15	0.24	0.08
**BP3**	-0.01	0.10	0.09	1.00	**0.82**	0.05	0.58	0.29	0.23	0.06	0.57	0.16	0.21
**4HB**	0.63	0.63	0.43	**0.82**	1.00	0.41	**0.67**	0.08	0.05	0.14	**0.75**	0.04	0.15
**4DHB**	-0.27	0.03	0.06	0.05	0.41	1.00	0.42	0.03	0.06	0.28	0.36	0.31	0.21
**AVO**	0.43	0.73	0.32	0.58	**0.67**	0.42	1.00	**0.67**	0.21	0.09	0.11	0.15	0.37
**EtPABA**	-0.31	0.38	0.12	0.29	0.08	0.03	**0.67**	1.00	0.23	0.11	0.09	0.13	0.44
**ODPABA**	-0.42	0.04	0.06	0.23	0.05	0.06	0.21	0.23	1.00	0.18	0.28	0.17	0.04
**EHMC**	0.05	0.19	0.09	0.06	0.14	0.28	0.09	0.11	0.18	1.00	0.01	0.09	0.17
**TBHPBT**	0.63	0.08	0.15	0.57	**0.75**	0.36	0.11	0.09	0.23	0.01	1.00	0.06	0.29
**UVP**	0.44	0.21	0.24	0.16	0.04	0.31	0.15	0.13	0.17	0.09	0.06	1.00	0.38
**UV328**	**0.93**	0.19	0.08	0.21	0.15	0.21	0.37	0.44	0.04	0.17	0.29	0.38	1.00
